# Draft Genome Sequences of 12 Dry-Heat-Resistant Bacillus Strains Isolated from the Cleanrooms Where the Viking Spacecraft Were Assembled

**DOI:** 10.1128/genomeA.00094-18

**Published:** 2018-03-22

**Authors:** Arman Seuylemezian, Kerry Cooper, Wayne Schubert, Parag Vaishampayan

**Affiliations:** a Biotechnology and Planetary Protection Group, California Institute of Technology, Jet Propulsion Laboratory, Pasadena, California, USA; b College of Agriculture and Life Sciences, School of Animal and Comparative Biomedical Sciences, The University of Arizona, Tucson, Arizona, USA

## Abstract

Spore-forming microorganisms are of concern for forward contamination because they can survive harsh interplanetary travel. Here, we report the draft genome sequences of 12 spore-forming strains isolated from the Manned Spacecraft Operations Building (MSOB) and the Vehicle Assembly Building (VAB) in Cape Canaveral, FL, where the Viking spacecraft were assembled.

## GENOME ANNOUNCEMENT

The Viking 1 and 2 spacecraft were launched in August and September 1975, respectively, to explore the planet Mars and specifically look for signs of extant extraterrestrial life (1). As part of a routine environmental microbial monitoring of the Vehicle Assembly Building (VAB) and the Manned Spacecraft Operations Building (MSOB) facilities in Cape Canaveral, FL, 32 Teflon ribbons were left out for 7 days and used to collect airborne microorganisms (2). The Teflon ribbons were then exposed to a total of 6 different heat treatments at three different time cycles (2).

Strains were sequenced on the Illumina HiSeq 2500 platform using a paired-end module. The CLC Genomics Workbench (version 10.1.1) was used to filter for adapter-free high-quality reads, which were subsequently *de novo* assembled. The draft genome statistics for all 12 strains are provided in Table 1. Draft genomes were annotated using both the NCBI Prokaryotic Genome Annotation Pipeline (PGAP) and the Rapid Annotations using Subsystems Technology (RAST) server.

**TABLE 1:** Draft genome statistics of 12 bacterial strains isolated from the VAB and MSOB facilities of the Kennedy Space Center during assembly of the Viking spacecraft

Strain	Taxonomic identification	GenBank accession no.	No. of contigs	Genome size (bp)	*N*_50_ (bp)	Largest contig (bp)	G+C content (%)	No. of 5S/16S/ 23S rRNAs	No. of protein- coding genes	Coverage (×)	No. of filtered reads
V32-6	Bacillus cucumis	PGVE00000000	110	5,707,899	158,423	338,809	38.61	6/3/1	5,309	142	5,418,558
M6-12	Bacillus sp.^*a*^	PGVF00000000	42	5,304,653	260,613	739,256	39.53	4/4/1	5,203	166	5,890,782
V3-13	Bacillus sp.^*a*^	PGUZ00000000	109	4,603,859	100,463	164,973	42.23	3/1/1	4,299	246	7,580,694
V33-4	Bacillus sp.^*a*^	PGVC00000000	98	4,350,768	90,863	192,558	41.83	5/1/2	4,096	293	8,526,302
T33-2	Bacillus sp.^*a*^	PGVB00000000	66	4,786,686	152,536	549,933	43.26	1/3/2	4,582	308	9,831,100
V48-19	Bacillus halotolerans	PGUV00000000	31	4,161,687	393,825	526,719	43.5	2/1/2	4,109	460	12,784,788
V5-8f	Bacillus sp.^*a*^	PGUW00000000	29	4,433,545	331,031	737,412	40.81	4/3/1	4,123	480	14,213,734
V21-33	Bacillus safensis	PGU00000000	14	3,715,075	882,133	1,006,594	41.59	3/2/1	3,730	571	14,151,324
V44_23b	Bacillus halotolerans	PEOF00000000	35	4,102,525	279,851	599,848	43.7	1/2/1	4,026	440	12,035,058
V1-29	Bacillus deserti	PGUY00000000	106	4,915,484	73,500	237,457	41.11	5/3/1	4,463	361	11,842,962
V16-21-2	Bacillus licheniformis	PIJD00000000	28	4,166,736	404,327	604,429	46.12	2/1/1	4,191	107	2,991,160
ATCC 29669	Bacillus canaveralius	PGVD00000000	117	4,694,986	74,302	324,486	41.64	4/1/5	4,399	562	17,615,760

a Potentially novel species of the genus Bacillus.

Strain V3-13 had putative genes coding for clustered regularly interspaced short palindromic repeat (CRISPR)-associated proteins Csn1 and Cas2. Strain V1-29 had genes coding for cobalamin synthase and lipoteichoic acid primase (LtaP), which are essential in the lipoteichoic acid synthesis pathway (3). Strain T33-2 had genes coding for prolyl peptidase, RNase E inhibitor RraA, which has been shown to affect the abundance of over 700 transcripts in Escherichia coli (4), and macrolide-specific efflux pump MacA. Strain V33-4 had genes coding for programmed cell death toxin MazF, which is part of the toxin-antitoxin system that allows for growth regulation under stressful conditions (5). Strain M6-12 had a putative RecX gene that functions in the SOS response and is typically coexpressed with RecA (6, 7). Strain V32-6 had genes coding for the HtrA protease/chaperone protein that plays a critical role in protein quality control (8), as well as a hydrogen peroxide-induced gene activator.

Strain V44_23b had a uniquely present gene coding for a phage tail protein and shared 41 genes with strain V48-19 only, such as the competence protein CoiA, which has roles in maintaining transformation efficiency (9), and the multidrug efflux transporter MdtP.

Strain V48-19 had genes coding for GgaA and GgaB that have roles in pathogenesis and antibiotic resistance (3, 10, 11) and have been shown to protect cells from thermally induced damage (12).

Strain ATCC 29669 had a uniquely present gene coding for glycogen branching enzyme GH-57 archaeal type, which was previously isolated in a hyperthermophilic archaeon, Thermococcus kodakaraensis KOD1^T^ (13). It also has genes coding for the cobalamin biosynthesis protein BluB, which was previously only thought to be present in Bacillus megaterium (14), and the biotin synthesis protein BioC.

Strain V21-33 had genes coding for phytoene desaturase that is involved in carotenoid biosynthesis (15), as well as spore germination proteins GerQB, GerHA/GerIA, and GerQC. Strain V5-8f had genes coding for a pseudouridine synthase (YciL), triacylglycerol lipases, and legionaminic acid cytidlyltransferase, which have roles in the biosynthesis of sialic acid (16). Strain V16-21-2 had unique genes coding for a chitodextrinase precursor that was previously identified in Vibrio furnissii (17) and an acetoin diacetyl reductase (18, 19).

### Accession number(s).

The genome sequences of all 12 isolates have been deposited at DDBJ/EMBL/GenBank under the accession numbers listed in Table 1.
